# Morphological and Molecular Identification of Mullet Helminth Parasite Fauna from Ganzirri Lagoon (Sicily, Southern Italy)

**DOI:** 10.3390/ani13050847

**Published:** 2023-02-25

**Authors:** Giovanni De Benedetto, Fabiano Capparucci, Sabrina Natale, Serena Savoca, Kristian Riolo, Claudio Gervasi, Marco Albano, Alessia Giannetto, Gabriella Gaglio, Carmelo Iaria

**Affiliations:** 1 Department of Veterinary Sciences, University of Messina, 98168 Messina, Italy; 2 Department of Chemical, Biological, Pharmaceutical and Environmental Sciences, University of Messina, 98166 Messina, Italy; 3 Department of Biomedical and Dental Sciences and Morphofunctional Imaging, University of Messina, 98166 Messina, Italy

**Keywords:** Acanthocephalan, trematode, *Chelon labrosus*, *Chelon auratus*, *Oedalechilus labeo*

## Abstract

**Simple Summary:**

We investigated the role of mullet as a biological tag to evaluate environmental status, mainly in a specific and circumscribed area, between March and June 2021, by conducting a survey of 150 mullet caught from Ganzirri Lagoon (Messina, Sicily, Italy), in order to describe the helminth fauna. All retrieved parasite specimens were investigated by morphological and molecular analysis. Morphological evaluation allowed us to describe the Acanthocephalan *Neoechinorhynchus agilis*; however, identification of the digenean trematode *Haploporus benedeni* was only possible using molecular analysis. None of the parasite species isolated in the present study presents a risk to human health.

**Abstract:**

Mullets (Osteichthyes: Mugilidae) are a euryhaline species widely distributed all over the world, thus representing an excellent study model for host–parasite interactions. From March to June 2022, 150 mullets, belonging to *Chelon labrosus* (*n* = 99), *Chelon auratus* (*n* = 37), and *Oedalechilus labeo* (*n* = 14) species, were caught to identify the helminth parasite fauna of the different mullet species present in the Ganzirri Lagoon (Messina, Sicily, Italy). A parasitological evaluation of the gastrointestinal tract (GIT) was carried out with a total worm count technique (TWC) to detect helminth presence. All collected parasites were stored in 70% ethanol until morphological evaluation, and frozen at −80 °C for subsequent molecular analysis, using 28S, ITS-2, 18S primers. The morphological evaluation allowed for the identification Acanthocephalan parasites (*Neoechinorhynchus agilis*) from two *C. labrosus* specimens. Sixty-six samples were positive for adult digenean trematodes (*C. labrosus*, 49.5 %; *C. auratus*, 27%, and *O. labeo*, 50%), molecularly identified as *Haploporus benedeni*. This study represents the first survey of helminthic parasite fauna of mullets from the south of Italy. The presence of *Hydrobia* sp. in the stomach contents of mullets allowed us to infer the *H. benedeni* life cycle in the Ganzirri lagoon.

## 1. Introduction

Fish are increasingly becoming the subjects of research studies, above all for their importance as a valuable source of protein. However, severe parasitic infections can cause functional disturbances, such as growth reduction and sensitivity to many secondary bacterial infections, as well as giving fish an unsightly appearance, thereby reducing their economic value. Fish populations play a key role in aquatic biological communities; moreover, they regulate the dynamics of the food chain, maintaining the balance of nutrients [1,2,3]. Thanks to the study of diet and migratory movements, fish fauna provide important data on anthropogenic action on all aquatic ecosystems [4,5], including reliable information on the possibility of accumulating pathogen organisms, such as bacteria, viruses, and parasites, as well as pollutants that pose a risk to human health [6,7].

Mugilidae, also known as mullets, are a large teleost family, divided into seventeen genera that include a high number of species [8,9,10,11,12,13]. They belong to the Actinopterygii and are characterized by a significant presence in shallow costal brackish waters, such as lagoons, rivers, estuaries, and lakes, between temperate, sub-tropical, and tropical areas, in both hemispheres [8]. They are also considered a secondary consumer, occupying an intermediate position in the food chain due to their eating habits [14]. In the Mediterranean Sea, four mullet genera (*Chelon, Liza*, *Mugil*, *Oedalechilus*) and six species (*Chelon labrosus*, *C. auratus*, *L. ramada*, *L. saliens*, *Mugil cephalus*, *O. labeo*) have been described [15]. Ganzirri Lagoon (Messina, Sicily, South Italy) is connected by several canals to the northern part of the Strait of Messina, forming two small ecosystems characterized by brackish water and high biodiversity levels in comparison with the other areas in the Strait, making it suitable for the exploitation of biological resources and for shellfish farming, an activity that has been practiced for several centuries. The lake is approximately 338,000 m^2^, 1.7 km long and 250 m wide, with a maximum depth of approximately 7 m and an average temperature that varies between 11° in January and 30° in August [16].

Mullet parasites associated with large-scale freshwater and seawater polyculture have been described worldwide since 1970 [17,18]. Mullets are economically important teleosts and are often farmed or caught for human consumption. However, like many species of fish, mullet can also be affected by parasites. Among the protozoan parasites that affect the Mugilidae, *Cryptocaryon irritans* [19,20], *Trichodina* sp. [21,22]. *Trichodinella* sp. [23], *Tetrahymena pyriformis*, and *T. pediculus* [24] have been reported globally. Dinoflagellata, *Amyloodinium ocellatum*, agent of the “velvet disease”, represents one of the most dangerous parasites in both wild and cultured mullets, causing high mortality rates [25,26]. Zoonotic Heterophidae metacercariae attributable to *Ascocotyle* sp., *Heterophyes heterophyes* and *Stictodora* sp., have been isolated from the muscle tissue of *C. labrosus* caught off the island of Sardinia and then morphologically [27] and molecularly identified [28]. The same parasite species have been isolated in *M. cephalus* from the Atlantic and Pacific Oceans [29] and in *L. ramada* from the Black Sea [30]. Monogenean flukes, such as *Microcotyle mugilis*, *Ligophorus* sp., and *Ergenstrema* sp. were found in *M. cephalus* sampled from the Marmara Sea (Turkey) [31] and *L. ramada* gills from Cabras Lagoon (Sardinia, Italy) [27,32]. Adult digenean trematodes, such as *Saccocoelium cephali*, *S. obesum, S. tensum,* and *Haploporus benedeni* were molecularly identified from *C. auratus* and *M. cephalus* gastrointestinal tracts caught along the Spanish Mediterranean coasts [33] and *L. ramada* (*p* = 36%) and *C. labrosus* (*p* = 67%) [27], where the Haploporidae was the most retrieved parasite family in all investigated species. *Neoechinorhynchus agilis* (Acanthocephala: Neoechinorhynchidae), which attaches to the intestinal submucosa layer, has mainly been observed in *M. cephalus* from the island of Sardinia (*p* = 91%) [27], and in other areas worldwide [34], as well as in other species such as *C. labrosus* and *C. auratus* [35]. Nematode infections in mullets are represented mainly by Anisakidae [36,37]. *Contracaecum* sp. larvae and *Capillaria* sp. have been described in Mugilids (*C. labrosus* and *C. auratus*) encysted in the celomic organ serosa and inside the gastrointestinal lumen [27], in the Mediterranean Sea, and particularly from the island of Sardinia.

The lack of information on the Mugilid gastrointestinal parasitic fauna in the study area is strictly related to the complexity of the environmental characteristics. Due to many variables, such as water movement and the prey present, it is difficult to improve current data on the control of parasites in wild fish. Thus, the aim of the current study is to characterize the gastrointestinal helminth parasite fauna of mullets from Ganzirri Lagoon (southern Italy).

## 2. Materials and Methods

### 2.1. Fish Sampling

From March to June 2021, during two different sampling trips in the same sampling site, a total of 150 mullet specimens were caught by a throwing net, also known as sparrow hawk, in the Nature Reserve of Ganzirri Lagoon (38°15′41″ N, 15°37′35″ E) (Messina, Sicily, Italy). 

After the sampling, all specimens were stored at +4 °C and transferred to the Experimental Fish Pathology Centre of Sicily (CISS), at the Department of Veterinary Sciences, University of Messina, Italy. All specimens were morphologically identified according to the key suggested by [8]. Fish were sexed, measured (total length, TL), and weighed (body weight, BW) (PBA220, Mettler Toledo, Columbus, OH, USA, accuracy of 1 g). All biometric data were recorded and subsequently reported as main values (mean length, ML; mean weight, MW). The specimens included in this manuscript were not part of any experiment and were commissioned by fish farmers for diagnostic purposes, specifically for fish disease control. For this reason, no ethical committee approval was needed, but all animal handling was, in any case, performed under European and Italian guidelines on animal welfare. All the sampling activities were authorized by the Regional Agency for the Protection of the Environment (ARPA Sicilia authorization n.1138/A of 15.03.2021).

### 2.2. Parasitological Examination

The evaluation of organs was carried out according to [38], modified according to requirements. The gastrointestinal (GI) tract was opened using scissors and the gastric and intestinal mucosa were gently scraped with the aid of a microscope slide; pyloric caeca were considered as part of the intestine; the organs were analyzed separately. GI contents and mucous were transferred into 1l graduated conical beakers and filled with saline solution for the subsequent sedimentation phase; the supernatant was replaced hourly to clarify the sediment. After 2 or 3 water changes, depending on supernatant clarity, the sediment was transferred to a Petri dish and monitored with the aid of a stereomicroscope (SteREO Discovery.V12 Zeiss, Jena, Germany). The collected parasite specimens were preserved in 70% ethanol until morphological identification. Other specimens were stored at −80 °C for subsequent molecular analyses. All retrieved specimens were diaphanized by immersion in glycerin for 24h, mounted and then identified with morphological keys [39,40,41]. Due to the unclear trematode identification by the morphological keys used, molecular analysis was performed only for these parasites. The morphological evaluation was performed under a light microscope (Axioskop 2 plus, Zeiss, Jena, Germany) and all pictures were taken using a digital camera (Axiocam Mrc, Zeiss, Jena, Germany) supported by a digital system (Axiovision, Zeiss, Jena, Germany). 

### 2.3. Molecular Analysis

Only the trematode specimens were used for molecular identification. After morphological evaluation and confirmation of the morphological characteristics, the samples were collected in three different pools and placed in 1.5 mL (Eppendorf, Hamburg, Germany); three series of 20 min washes were performed with a phosphate buffer saline (PBS) buffer solution. DNA was extracted using a Wizard SV Genomic DNA Purification System (Promega, Madison, WI, USA). Concentration and purity were verified using a Nanodrop spectrophotometer (Thermo Fisher Scientific, Waltham, MA, United States). The amplification of different rDNA regions was performed using a GoTaq^®^ Colorless Master Mix (Promega, Madison, WI, United States) and different primers for trematode identification: 28S, ITS-2 and 18S: forward primer 5′ GTCCGATAGCGAACAAGTACCGT 3′ and reverse primer 5′ AGCATAGTTCACCATCTTTCGGGTCTCAA 3′ for 28S; forward primer 5′ GTCGTAACAAGGTAGCTGTA 3′ and reverse primer 5′ TATGCTTAAGTTCAGCGGGT 3′ for the partial ITS-2 [41], while for the oligonucleotide primers, C-For ATGGCTCATTAAATCAGCTAT and A-Rev TGCTTTGAGCACTCAAATTTG were used for 18S amplification [42]. The thermal cycling conditions were the following: initial denaturation for 30 s at 94 °C, 35 cycles of denaturation for 30 s at 94 °C, annealing at 58 °C for 30 s, elongation for 60 s at 72 °C with final extension of 10 min at 72 °C for 28S rDNA; initial denaturation for 30 s at 94 °C, 35 cycles of denaturation for 30 s at 94 °C, annealing at 56 °C for 90 s, elongation for 90 s at 72 °C with final extension of 10 min at 72 °C for ITS 2; initial denaturation at 94 °C for 2 min, denaturation at 94 °C for 30 s, annealing at 56 °C for 30 s, elongation at 72 °C for 60 s repeated 30 cycles, followed by a final 60 s elongation at 72 °C for 18 s [42,43]. Positivity was assessed on 1% (*w***/***v*) agarose gel. Once the presence of bands had been ascertained, a gel extraction was carried out using the Promega kit Wizard^®^ SV Gel and PCR Clean-Up System. The concentration and purity were verified using the Nanodrop spectrophotometer (Thermo Fisher Scientific, Waltham, MA, USA) and samples were sent to Genechron Biotech for sequencing in both forward and reverse directions using the same primers used for amplification.

### 2.4. Statistical Analysis

Epidemiological infection indices of prevalence (*p*, %), mean abundance (MA), and mean intensity (MI) were calculated according to [44]. All data are presented as the mean ± standard deviation (SD). A chi-squared test was performed to assess parasite occurrence among investigated Mugilid species. The non-parametric Kruskal–Wallis’s test, followed by Dunn’s multiple comparisons test, were performed to highlight any significant difference in parasite abundance between districts and investigated Mugilid species. Furthermore, a Spearman correlation analysis was performed to detect any possible correlation between parasite abundance and Mugilid morphological features. The level of significance was set at *p* values < 0.05. A statistical analysis was performed using the software Prism V.9.0.0 (Graphpad Software Ldt., La Jolla, CA, USA).

## 3. Results

A total of 150 mullets were examined. Identification of Mugilidae sampled species revealed a heterogeneous group composed mainly of *C. labrosus* (99/150), followed by *C. auratus* (37/150) and *O. labeo* (14/150). All biometric ML and MW data on the three retrieved species are reported in Table 1. 

### 3.1. Parasitological Findings

**Figure 1:**
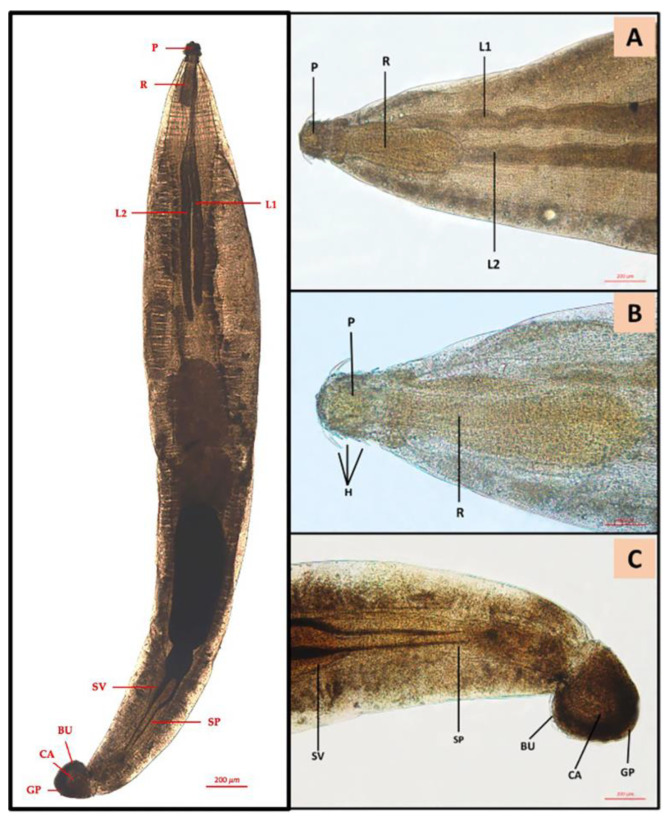
*Neoechinorhynchus agilis* cranial and posterior end: (**A**) proboscis (P), proboscis receptacle (R), lemnisci (L1, L2); (**B**) proboscis (P), proboscis receptacle (R), hook systems (H); (**C**) *N. agilis* male posterior end: seminal vesicle (SV), Saefftigen’s pouch (SP), bursa (BU), calotte (CA), genital pore (GP).

Of the 150 sampled fish GI tracts, 67 (44.7%) presented at least one parasite species. Sixty-six (44%) specimens were affected by adult digenean trematodes, both in stomach and gut. Of these, 2 *C. labrosus* specimens (1.3%) were affected by a total number of 16 Acanthocephala in the gut, identified according to [41] as *Neochinorhynchus agilis* (Neoechinorhynchidae, Rudolphi, 1819) (Figure 1).

Trematode loads per species are reported in Table 2.

**Figure 2:**
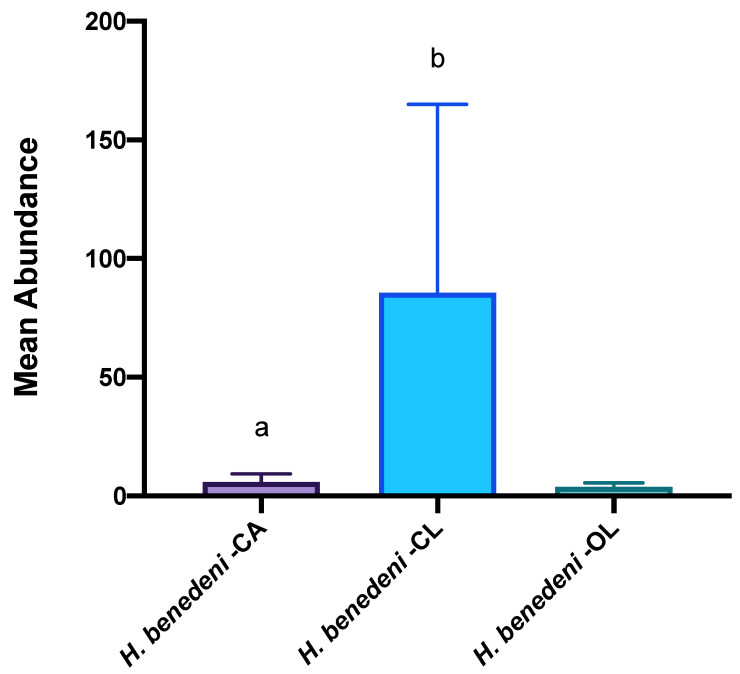
Mean abundance of *Haploporus benedeni* found in all three mullet species: CL: *Chelon labrosus*; CA: *Chelon auratus*; OL: *Oedalechilus labeo*. Each datum is shown as mean ± SD. Letters are only present in the case of significant statistical differences. Different letters refer to significant differences between different species. Differences were considered significant when *p* < 0.05.

The results of the chi-squared test did not show any significant difference in parasite occurrence between the three Mugilidae species (χ^2^ = 5.7; df = 2; *p* = 0.056). A different result was observed for the abundance data, which varied significantly between *C. auratus* and *C. labrosus* (*p* <0.05), as shown in Table 3 and Figure 2. No significant differences were found between districts (*p* > 0.05).

Finally, Spearman’s correlation was performed to detect any possible correlation between parasite abundance and morphometric fish features, i.e., total length (TL) and body weight (BW). The results did not show any significant correlation between parasite abundance detected in CA and CL with these parameters. However, OL parasite abundance was negatively correlated both to the TL (r = −0.68; *p* = 0.009) and BW (r = −0.64; *p* = 0.015) of the specimens.

### 3.2. Molecular Analysis

The nucleotide sequences of the amplified products were identical among biological replicates. The sequences obtained for 18S (727 bp)*,* ITS-2 (401 bp), and 28S (871 bp) were analyzed by BLAST similarity search against the National Center for Biotechnology Information (NCBI; https://blast.ncbi.nlm.nih.gov/Blast.cgi (accessed on 14 July 2022) database to calculate the statistical significance of the matches found.

In particular, BLAST analysis of the sequences obtained from ITS-2 showed a similarity of 99.5% (E-value 0.0; query cover 100%) with the relative sequence from *Haploporus benedeni* (accession number FJ211247.1) while there was a similarity of 100% for 28S (Accession number FJ211237.1) and 99.86% for 18S (accession number FJ211228); *Haploporus benedeni* (Stossich, 1887) was found with an E-value of 0.0 and a Query cover of 100% for both sequences. Amplification of the 28S rDNA gene included the D2 domain, the most robust domain, since it could provide a higher resolution of the species. The representative DNA sequence for 18S, ITS-2, and 28S was submitted to GenBank (accession numbers OQ283825, OQ283827, OQ283826, respectively).

## 4. Discussion

The present study represents the first survey on mullet helminth parasite fauna from a Special Protection Area (SPA) in the central Mediterranean Sea. Digenean parasites in mullets from different areas of the Mediterranean Sea have been reported [27,35]. Several digenean parasites were collected in the intestinal mucosa [45,46], as reported in *L. saliens* and *L. ramada* [45]. 

In the present study, the most abundant collected species was *C. labrosus*; nevertheless, the most parasitized species was *O. labeo*, although it is the least representative collected species and the high prevalence is probably related to a specific ecological characteristic of *O. labeo* to host the trematode herein described, localized mainly in the intestine, rather than the stomach.

Another relevant aspect of the investigated area, characterized by regular movement of water, is the difference in the salinity rate in comparison with the nearby sea. Salinity is strictly related to mollusk and crustacean evolution, regulating both growth and intermediate host vulnerability to the predators [16]. The likelihood that the sampling spot is distant from the infection point, even by a couple of kilometers, in an unknown area, characterized by different environmental conditions, should be highlighted [47].

The parasitological examination and subsequent molecular confirmation allowed us to identify the trematode *Haploporus benedeni* (Haploporidae, Stossich 1887) using the ITS2, 18S, and 28S ribosomal RNA genes. Despite the high number of reports of *H. benedeni* in most Mugilid species from the Mediterranean basin [48,49,50,51], few studies provide a molecular identification of this species [33]; therefore, our study improves the current knowledge about the molecular description of this digenean trematodes. The *H. benedeni* life cycle is characterized by the free-swimming miracidium and an intermediate host belonging to the genus *Hydrobia*, as reported by [52]. In particular, *Hydrobia* sp. is a small aquatic gastropod mollusk belonging to the family Hydrobiidae. The presence of *Hydrobia* sp. inside the mullet stomach could be associated to the *H. benedeni* prevalence in each of the three studied species, which also indicates a high distribution of snails belonging to the *Hydrobia* sp. in the investigated area. The *H. benedeni* prevalence reported by [27] in *C. labrosus* (67%) was higher than the present study, where the most infected species was *O. labeo*. *Neoechinorhynchus agilis* is a widespread parasite of grey mullets worldwide [27,41]. In [47], *N. agilis* specimens attached to the intestinal mucosal and submucosal layers of *L. saliens* and *L. ramada* (41.6%) at a higher prevalence than in the present study (1.3%). This Acanthocephalan has also usually been considered as a *M. cephalus* parasite [46]. *C. labrosus* was found in the eastern Atlantic Ocean and Mediterranean Sea as one of the most infected species (up to 54%) by *N. agilis* [27,41], a prevalence higher than our study (1.3%), which is probably related to the difference in fish size. The identified parasites, *H. benedeni* and *N. agilis*, previously reported from the Mistras Lagoon (Sardinia—Italy—western Mediterranean Sea) [14], were reported for the first time in the central Mediterranean Sea, showing an equal distribution in two different Mediterranean areas, as well as in Libya and Egypt [53]. This ecological evaluation is also supported by the current information on the diversity and distribution of parasites, such as *H. benedeni* and *N. agilis* in mullets. 

## 5. Conclusions

In conclusion, this study improves the current knowledge on mullet helminth parasitic fauna from the Ganzirri Lagoon in the central Mediterranean Sea. Mullet, while not characterized by a high commercial value, are increasingly being appreciated both for their greater production in aquaculture and for their use in traditional food preparation in the Mediterranean basin. *Hydrobia* spp., a common mollusk found in Ganzirri Lake, and considered a host for a wide range of trematode species that can infect several marine species such as mullet, can be used as a biological tag for further studies in the same area. Moreover, even if mullets are considered a host of parasites which are potentially dangerous for human health, the parasitic species identified in the examined GIT are not considered zoonotic agents.

## Figures and Tables

**Table 1:** Mean lengths, mean weights, and standard deviations (SD) of the three species.

Species	Mean Length (cm) ± SD	Mean Weight (g) ± SD
*Chelon labrosus*	20.18 ± 3.99	91.66 ± 139.67
*Chelon auratus*	19.75 ± 1.9	72.08 ± 22.19
*Oedalechilus labeo*	18.75 ± 2.74	65.28 ± 23.97

**Table 2:** Prevalence, intensity, and abundance of *Haploporus benedeni* in the three species studied. S—Stomach; I—Intestine; GIT—Gastro-intestinal tract.

***Chelon labrosus***			
	S	I	GIT
Prevalence %	12.12	14.14	24.24
Mean intensity	6.16	7.78	346.3
Mean abundance	0.74	1.1	83.9
***Chelon auratus***			
	S	I	GIT
Prevalence %	2.7	16.2	8.1
Mean intensity	2	7	57.6
Mean abundance	0.05	1.13	4.67
***Oedalechilus labeo***			
	S	I	GIT
Prevalence %	50	-	50
Mean intensity	8.66	-	6.5
Mean abundance	1.85	-	1.85

**Table 3:** Results of the Kruskal–Wallis test and Dunn’s multiple comparison test of the *Haploporus benedeni* abundances in the three mullet species.

**Kruskal–Wallis Test**			
*p* value	0.0374		
*p* value summary	*		
Do the medians vary signif. (*p* < 0.05)?	Yes		
Number of groups	3		
Kruskal–Wallis statistic	6.573		
**Dunn’s multiple comparisons test**	**Mean rank diff.**	**Summary**	**Adjusted *p* Value**
*H. benedeni* -CA vs. *H. benedeni* -CL	−19.29	*	0.0343
*H. benedeni* -CA vs. *H. benedeni* -OL	−18.69	ns	0.397
*H. benedeni* -CL vs. *H. benedeni* -OL	0.5988	ns	>0.9999

* *p* value ≤ 0.05

## Data Availability

Not applicable.
